# Reply: ATP10B variants in Parkinson’s disease—a large cohort study in Chinese mainland population

**DOI:** 10.1007/s00401-021-02281-8

**Published:** 2021-02-18

**Authors:** Stefanie Smolders, Christine Van Broeckhoven

**Affiliations:** Neurodegenerative Brain Diseases, Department of Biomedical Sciences, VIB Center for Molecular Neurology, University of Antwerp, Universiteitsplein 1, 2610 Antwerp, Belgium

We appreciate that Zhao et al. aimed at replicating our finding of homozygous and compound heterozygous rare variants in *ATP10B*, increasing the risk for Parkinson's disease (PD) by compromising lysosomal glucosylceramide export [3]. In a large cohort of PD patients from the Chinese mainland population, they identified one early-onset PD homozygous carrier and five early-onset PD carries of compound heterozygous *ATP10B* rare variants (minor allele frequency ≤ 1%) [8]. They also observed two compound heterozygous carriers of rare *ATP10B* variants in the control group. Li et al. identified three carriers of compound heterozygous *ATP10B* rare variants in another Chinese cohort consisting of 743 unrelated early-onset PD patients [1]. The *cis*/*trans* phase of these compound heterozygous *ATP10B* carriers could not be determined in both studies [1, 8]. Li et al. identified also identified a PD carrier of the p.G671R/p.N865K rare variants, which are most likely in *cis* configuration as demonstrated in our Belgian study [1, 3]. The Belgian cohorts in our study comprised 617 unrelated PD patients and 598 unrelated control individuals [3]. Combinations of rare *ATP10B* variants in compound heterozygous carriers (frequency < 0.01%) are associated with an increased risk for PD. Because the Belgian cohort sizes are limited, we need replication studies of our findings in independent and larger cohorts to estimate a more accurate risk contribution of the homozygous and compound heterozygous *ATP10B* rare variants to PD etiology. Since the publication of our initial discovery, others have attempted to replicate our genetic findings. Real et al. identified 1.9% of PD patients (4.8% in early-onset PD patients) and 2.0% of control carriers of compound heterozygous *ATP10B* variants in large PD patient and control cohorts of European ancestry [4]. In this study, the phase of the compound heterozygous variants of the carriers is also unknown [4, 5]. Tesson et al. investigated the presence and segregation of bi-allelic *ATP10B* variants in 17 autosomal recessive PD families originating from Europe, North Africa and Turkey [7]. They identified a PD carrier homozygous for 3 *ATP10B* variants and developing disease 11 years earlier than his affected sibling compound heterozygous for these *ATP10B* variants in *cis* configuration. This observation queries whether recessive *ATP10B* alleles might have a modifying role on age at onset [6, 7].

As demonstrated by the studies of Zhao et al., Li et al. and Real et al., the genetic evidence for autosomal recessive inherited mutations in *ATP10B* is limited due to difficulties in *cis*/*trans* phasing of the compound heterozygous mutations [1, 4, 8]. Family-based studies, such as the study of Tesson et al., have the advantage of relatives for phasing compound heterozygous variants [7]. But, 17 families are not sufficient to replicate the association of recessive *ATP10B* variants with increased PD risk [6]. To overcome this hurdle, there is an urgent need for family trio studies or optimized methods for phasing compound heterozygous variants identified in large cohorts of unrelated patients or control individuals. Unfortunately, phasing of variants separated by a long genomic distance is not possible with short-read sequencing technologies, which are often used in large cohort studies. Long-read sequencing technologies are able to overcome this obstacle if the genomic distance of the variants is acceptable or if long-read sequencing can be performed on cDNA. The latter demands the availability of cells or tissue in which the gene is expressed. Expression of *ATP10B* is limited to the gastrointestinal system and brain, which limits cDNA analysis [3]. To detect compound heterozygous single-nucleotide variants in long-read sequencing data, optimized methods are needed. Currently, long-read data is less accurate for the detection of single-nucleotide variants compared to short-read sequencing data [2].

Another factor limiting genetic evidence of *ATP10B* is the presence in control carriers of rare putative compound heterozygous *ATP10B* variants. Usually, before the inclusion of individuals in the control group they are screened for neurological or psychiatric antecedents and neurological complaints. However, generally control individuals are not followed-up and neurological diseases might appear later, particularly when the controls were included at an early age. The mean ages at inclusion (AAI) of the controls in our discovery study, the replication study of Real et al. and the replication study of Zhao et al. were 70.4 ± 7.9 years, 60.3 ± 11.9 years and 54.3 ± 13.1 years [3, 4, 8]. The age at onset (AAO) of the PD patients carrying compound heterozygous *ATP10B* variants varied between 24 to 68 years in our study [3]. This high variability in AAO makes it difficult to estimate the pathogenicity of compound heterozygous variants in seemingly healthy control individuals who are still at risk of developing the disease. Variable effects of single mutant alleles on ATP10B protein expression and/or functioning may explain the variability in AAO and the presence of compound heterozygous mutations in control individuals [3]. Therefore, functional validation of rare homozygous and compound heterozygous *ATP10B* variants in both patient and control carriers is equally important to estimate their contribution to PD.

Overall, we have to conclude that all replication studies reported to date were unable to replicate our findings but could not exclude a potential role of rare homozygous and *trans* compound heterozygous *ATP10B* variants to PD risk. Recessive *ATP10B* variants are rare and therefore *ATP10B* screening in large cohorts, combined with *cis/trans* phasing of compound heterozygous variants, neurological follow-up of control carriers and functional experiments are of utmost importance.
